# Supplementary material for: Unveiling the Bioactive Potential of the Invasive Jellyfish Phyllorhiza punctata Through Integrative Transcriptomic and Proteomic Analyses
Source: Biomolecules. 2025 Aug 4;15(8):1121. doi: 10.3390/biom15081121 (PMC12383608; doi:10.3390/biom15081121)
Supplement: Supplementary file 1 [file biomolecules-15-01121-s001.zip › Suplementary Material/FileS3/PEPFOLD4/XP_065059049_4552/clusters.html]

 PEP-FOLD clusters


Download archive of all models  here   

|  |  |  |
| --- | --- | --- |
| # | file | sOPEP |
| # cluster 1 - Size: 8 |  |  |
|  | XP\_065059049\_4552-model1 | -0.914606 |
|  | XP\_065059049\_4552-model1.1 | -0.914606 |
|  | XP\_065059049\_4552-model1.2 | -0.903116 |
|  | XP\_065059049\_4552-model1.3 | -0.88348 |
|  | XP\_065059049\_4552-model1.4 | -0.88348 |
|  | XP\_065059049\_4552-model1.5 | -0.868832 |
|  | XP\_065059049\_4552-model1.6 | -0.846518 |
|  | XP\_065059049\_4552-model1.7 | -0.818687 |
| # cluster 2 - Size: 3 |  |  |
|  | XP\_065059049\_4552-model2 | -0.911099 |
|  | XP\_065059049\_4552-model2.1 | -0.890086 |
|  | XP\_065059049\_4552-model2.2 | -0.890086 |
| # cluster 3 - Size: 5 |  |  |
|  | XP\_065059049\_4552-model3 | -0.900828 |
|  | XP\_065059049\_4552-model3.1 | -0.857653 |
|  | XP\_065059049\_4552-model3.2 | -0.857653 |
|  | XP\_065059049\_4552-model3.3 | -0.850689 |
|  | XP\_065059049\_4552-model3.4 | -0.796727 |
| # cluster 4 - Size: 5 |  |  |
|  | XP\_065059049\_4552-model4 | -0.886492 |
|  | XP\_065059049\_4552-model4.1 | -0.886492 |
|  | XP\_065059049\_4552-model4.2 | -0.886492 |
|  | XP\_065059049\_4552-model4.3 | -0.886492 |
|  | XP\_065059049\_4552-model4.4 | -0.840895 |
| # cluster 5 - Size: 9 |  |  |
|  | XP\_065059049\_4552-model5 | -0.874206 |
|  | XP\_065059049\_4552-model5.1 | -0.874206 |
|  | XP\_065059049\_4552-model5.2 | -0.874206 |
|  | XP\_065059049\_4552-model5.3 | -0.871818 |
|  | XP\_065059049\_4552-model5.4 | -0.864748 |
|  | XP\_065059049\_4552-model5.5 | -0.864748 |
|  | XP\_065059049\_4552-model5.6 | -0.864748 |
|  | XP\_065059049\_4552-model5.7 | -0.864748 |
|  | XP\_065059049\_4552-model5.8 | -0.811224 |
| # cluster 6 - Size: 5 |  |  |
|  | XP\_065059049\_4552-model6 | -0.871861 |
|  | XP\_065059049\_4552-model6.1 | -0.868116 |
|  | XP\_065059049\_4552-model6.2 | -0.866032 |
|  | XP\_065059049\_4552-model6.3 | -0.857139 |
|  | XP\_065059049\_4552-model6.4 | -0.827473 |
| # cluster 7 - Size: 3 |  |  |
|  | XP\_065059049\_4552-model7 | -0.870996 |
|  | XP\_065059049\_4552-model7.1 | -0.859966 |
|  | XP\_065059049\_4552-model7.2 | -0.844199 |
| # cluster 8 - Size: 23 |  |  |
|  | XP\_065059049\_4552-model8 | -0.869568 |
|  | XP\_065059049\_4552-model8.1 | -0.864395 |
|  | XP\_065059049\_4552-model8.2 | -0.864216 |
|  | XP\_065059049\_4552-model8.3 | -0.864216 |
|  | XP\_065059049\_4552-model8.4 | -0.864216 |
|  | XP\_065059049\_4552-model8.5 | -0.864216 |
|  | XP\_065059049\_4552-model8.6 | -0.864216 |
|  | XP\_065059049\_4552-model8.7 | -0.863855 |
|  | XP\_065059049\_4552-model8.8 | -0.863855 |
|  | XP\_065059049\_4552-model8.9 | -0.863855 |
|  | XP\_065059049\_4552-model8.10 | -0.859127 |
|  | XP\_065059049\_4552-model8.11 | -0.859127 |
|  | XP\_065059049\_4552-model8.12 | -0.859127 |
|  | XP\_065059049\_4552-model8.13 | -0.857678 |
|  | XP\_065059049\_4552-model8.14 | -0.857678 |
|  | XP\_065059049\_4552-model8.15 | -0.855499 |
|  | XP\_065059049\_4552-model8.16 | -0.853244 |
|  | XP\_065059049\_4552-model8.17 | -0.853244 |
|  | XP\_065059049\_4552-model8.18 | -0.850331 |
|  | XP\_065059049\_4552-model8.19 | -0.850233 |
|  | XP\_065059049\_4552-model8.20 | -0.849728 |
|  | XP\_065059049\_4552-model8.21 | -0.845575 |
|  | XP\_065059049\_4552-model8.22 | -0.797435 |
| # cluster 9 - Size: 8 |  |  |
|  | XP\_065059049\_4552-model9 | -0.863576 |
|  | XP\_065059049\_4552-model9.1 | -0.863402 |
|  | XP\_065059049\_4552-model9.2 | -0.861331 |
|  | XP\_065059049\_4552-model9.3 | -0.855549 |
|  | XP\_065059049\_4552-model9.4 | -0.826554 |
|  | XP\_065059049\_4552-model9.5 | -0.806702 |
|  | XP\_065059049\_4552-model9.6 | -0.791555 |
|  | XP\_065059049\_4552-model9.7 | -0.788239 |
| # cluster 10 - Size: 4 |  |  |
|  | XP\_065059049\_4552-model10 | -0.862018 |
|  | XP\_065059049\_4552-model10.1 | -0.862018 |
|  | XP\_065059049\_4552-model10.2 | -0.859938 |
|  | XP\_065059049\_4552-model10.3 | -0.859938 |
| # cluster 11 - Size: 7 |  |  |
|  | XP\_065059049\_4552-model11 | -0.85875 |
|  | XP\_065059049\_4552-model11.1 | -0.853411 |
|  | XP\_065059049\_4552-model11.2 | -0.853411 |
|  | XP\_065059049\_4552-model11.3 | -0.850199 |
|  | XP\_065059049\_4552-model11.4 | -0.846111 |
|  | XP\_065059049\_4552-model11.5 | -0.846111 |
|  | XP\_065059049\_4552-model11.6 | -0.83361 |
| # cluster 12 - Size: 3 |  |  |
|  | XP\_065059049\_4552-model12 | -0.857565 |
|  | XP\_065059049\_4552-model12.1 | -0.857565 |
|  | XP\_065059049\_4552-model12.2 | -0.84238 |
| # cluster 13 - Size: 6 |  |  |
|  | XP\_065059049\_4552-model13 | -0.830968 |
|  | XP\_065059049\_4552-model13.1 | -0.830968 |
|  | XP\_065059049\_4552-model13.2 | -0.830968 |
|  | XP\_065059049\_4552-model13.3 | -0.8079 |
|  | XP\_065059049\_4552-model13.4 | -0.806043 |
|  | XP\_065059049\_4552-model13.5 | -0.806043 |
| # cluster 14 - Size: 10 |  |  |
|  | XP\_065059049\_4552-model14 | -0.803493 |
|  | XP\_065059049\_4552-model14.1 | -0.803493 |
|  | XP\_065059049\_4552-model14.2 | -0.803493 |
|  | XP\_065059049\_4552-model14.3 | -0.803493 |
|  | XP\_065059049\_4552-model14.4 | -0.803493 |
|  | XP\_065059049\_4552-model14.5 | -0.803493 |
|  | XP\_065059049\_4552-model14.6 | -0.803493 |
|  | XP\_065059049\_4552-model14.7 | -0.803493 |
|  | XP\_065059049\_4552-model14.8 | -0.78619 |
|  | XP\_065059049\_4552-model14.9 | -0.775853 |
| # cluster 15 - Size: 1 |  |  |
|  | XP\_065059049\_4552-model15 | -0.782301 |
|  |  |  |
